# Palpation as a Method To Predict Spatial Instrumental Hyolaryngeal Excursion Measures

**DOI:** 10.1007/s00455-025-10880-w

**Published:** 2025-09-25

**Authors:** Kelsey L. Murray, Sarah H. Szynkiewicz, Erin Kamarunas

**Affiliations:** 1 School of Medicine, Department of Otolaryngology, University Mississippi Medical Center, Jackson, MS USA; 2 School of Health Professions, Department of Communication Sciences and Disorders, Samford University, Birmingham, AL USA; 3 Department of Communication Sciences and Disorders, James Madison University, Harrisonburg, VA USA; 4 Sentara Rockingham Memorial Hospital, Harrisonburg, VA USA

**Keywords:** Hyolaryngeal excursion, Clinical swallow evaluation, Dysphagia, Palpation

## Abstract

The purpose of this study is to determine if speech-language pathologists’ (SLP) ratings of palpated hyolaryngeal excursion (pHLE) during a clinical swallow evaluation (CSE) are predictive of spatial measures of hyolaryngeal excursion determined by instrumentation (iHLE). Adults between the ages of 18–99 were recruited with a physician referral to complete a CSE and videofluoroscopy swallow study. Four SLP investigators completed ratings of pHLE palpation during a CSE. Spatial measures of hyoid peak elevation were taken from videofluoroscopy swallow studies. Statistical analyses included multiple linear regression to determine the best-fitting model to predict iHLE from palpated ratings. Data from 77 volunteers (44 female, mean age 71.6) were used for statistical analyses. The linear regression model indicated three significant predictors of superior (upward) hyoid peak position, including palpation, bolus consistency, and the number of swallows. There were no significant predictors of anterior (forward) hyoid peak position from the tested factors. The emergence of these significant predictors suggests that palpation may provide insight into superior HLE movements during a CSE. Next steps will be to determine if palpation improves SLPs’ diagnostic accuracy and clinical decision-making during swallow assessment without instrumentation.

**Supplementary Information:**

The online version contains supplementary material available at 10.1007/s00455-025-10880-w.

 Dysphagia is a disorder impacting swallowing function in the oral cavity, pharynx, and/or esophagus that can lead to serious medical consequences such as dehydration, malnutrition, aspiration pneumonia, or other cardiorespiratory events [1]. One in six adults in the United States reports having symptoms of dysphagia [2]. Hyolaryngeal excursion (HLE) is a crucial aspect of swallowing safety that involves the anterior and superior movement of the hyoid bone and the connected larynx at the onset of swallowing, driven by the contraction of suprahyoid muscles [3]. Specifically, the mylohyoid, posterior belly of the digastric, and stylohyoid muscles facilitate elevation of the hyolaryngeal unit and the anterior belly of the digastric and geniohyoid muscles pull the hyoid and larynx forward [4]. Figure 1 illustrates HLE trajectory from resting to the highest peak with the labeled x and y lines representing anterior and superior movement, respectively. Hyolaryngeal excursion is vital for airway protection, facilitating epiglottic inversion and the closure of the laryngeal vestibule to prevent penetration or aspiration into the airway [5–8]. HLE also enhances swallowing efficiency by stretching the cricopharyngeus muscle, which contributes to pharyngoesophageal segment (PES) opening, allowing the bolus to pass into the esophagus [5–7, 9, 10].

Speech-language pathologists (SLP) typically use the clinical swallow evaluation (CSE) as their first course of action in evaluating dysphagia [1, 11–14]. The CSE includes a case history review, patient/family interviews, cognitive-communication/language screen, cranial nerve/oral motor exam, and swallowing trials as warranted [1, 13, 15, 16]. The CSE relies heavily on the subjective judgment of the SLP’s perceived visual, tactile, and auditory interpretations of oropharyngeal events to detect signs of dysphagia [16–19].

## Assessment and Purpose of HLE during a CSE

Observation of HLE is often included as a component of the CSE and is typically assessed as the patient is swallowing saliva, food, or drink using perceptual-based ratings, including laryngeal palpation, visual observation of the thyroid notch, and observed signs/symptoms (e.g., vocal quality changes [20]. Palpation of HLE (pHLE) involves the SLP placing their fingers along the external anterior neck in the submental and laryngeal regions to detect laryngeal movement associated with swallowing [21] (Fig. 2). A previous study demonstrated movements of the hyoid and larynx are positively correlated in both the horizontal and vertical planes via instrumental assessment [22] and therefore, palpated laryngeal movement via the thyroid notch is considered a sufficient substitute for movement of hyolaryngeal complex. It has been suggested that this technique, combined with observation of thyroid notch movement, provides SLPs valuable insights into hyolaryngeal movement and timing, allows the SLP to count the number of swallows completed and helps estimate oral transit time [21].

### Potential Limitations of pHLE Assessment Via Palpation

While the CSE often includes judgments of pHLE, a detailed description or methodology of how it was assessed has not always been provided, which highlights a gap in evidence-based guidance for SLPs [11, 13, 23–25]. The Mann Assessment of Swallowing Assessment (MASA) is a standardized CSE that includes instructions for assessing HLE through palpation of the thyroid notch with four fingers, as referenced in Logemann [21] and rating it on a four-point scale [26]. Subjective rating of the extent of hyolaryngeal movement is required (e.g., mildly restricted, incomplete) and may be challenging, especially for subtle reductions. SLPs often lack preexisting knowledge of patients’ premorbid HLE, making it difficult to judge potential changes in movement. SLPs should also be aware that external and internal variables can impact HLE range of motion, which may affect how pHLE is judged [17]. Patient factors such as age, sex, and effort may impact the extent of hyolaryngeal movement, while bolus factors such as volume, consistency, temperature, and taste likely also affect HLE movement [27, 28].

In addition to the lack of a well-defined assessment protocol for pHLE, it is unclear if palpation during swallowing is diagnostically informative about the pharyngeal swallowing phase [17]. Perhaps for this reason, reported use of palpation during the CSE seems to be in decline over the past 25 years. The reported frequency of hyolaryngeal elevation assessment during clinical swallow evaluation has continued to decline from 95.8% in 1999 to 89.9% in 2018 and, more recently in 2022, at 63% [15, 20, 29]. However, palpation as a method to assess laryngeal elevation during a CSE has fluctuated, the first two studies only included palpation using Logemann’s 4-finger method which decreased from 62.1% in 1999 to 46.1% in 2018 [15, 20, 29], however, a recent study determined that pHLE is used 57% including palpating using a variation number of fingers with 2–3 fingers primary purpose [20] demonstrating that the 4-finger method may have had an increase decline over pHLE, during a CSE. A relatively new finding is that some SLPs do not complete HLE assessment as a component of the CSE [20, 29]. Despite the apparent decline in use, SLPs are assessing HLE in some form in the majority, highlighting the necessity of determining best practices for pHLE assessment.

A recent retrospective study used electronic medical record documentation of clinical HLE judgments to compare against spatial measures of superior and anterior hyolaryngeal excursion (referenced hereafter as instrumental hyolaryngeal excursion, iHLE) obtained from videofluoroscopy swallow studies (VFSS) [17]. Patients whose record documented reduced pHLE during the CSE were found to have significantly less *superior* iHLE movement compared to those documented to have normal pHLE. However, this difference was not present for anterior iHLE. This suggests that clinically assessed HLE may effectively detect reduced *superior* hyolaryngeal movement but may not be as useful for interpreting anterior hyolaryngeal movement.

While these findings are promising, the inherent limitations of the retrospective design warrant caution in over-interpreting the results. Controlled prospective work is critical to confirm the utility of pHLE. This study aims to determine whether ratings of palpated HLE during a CSE can predict instrumentally determined spatial HLE measures (iHLE). This is the first prospective study to compare perceptual ratings of HLE palpation with spatial measures confirmed with instrumentation, determining the diagnostic value of palpation in a systematic and controlled manner.

## Methods

### Patients

The study was approved by James Madison University’s and Sentara Rockingham Memorial Hospital’s Institutional Review Boards. Patients were recruited from inpatient and outpatient clinics. Patients over the age of 18 years were eligible if they were referred for a videofluoroscopic swallow study (VFSS) by a physician due to swallowing difficulty reported by the patient or a healthcare provider (e.g., physician, nurse, speech-language pathologist (SLP). Inclusion criteria included a current VFSS order, orientation to self, cognitive ability to follow a one-step direction, and ability to sign the consent form. Exclusion criteria included a history of supraglottic laryngectomy or total laryngectomy surgery that would make spatial hyolaryngeal excursion measures determined by instrumentation (iHLE) difficult or not possible.

### SLPs’ Experience and Setting

Four licensed and certified speech-language pathologists served as raters in this study. The SLPs had an average of 8.75 years of clinical experience (range: 3–16) in inpatient acute care, outpatient, and/or skilled nursing settings.

## Setup and Procedures

### Clinical Swallow Evaluation (CSE) Setup and Procedure

All palpated hyolaryngeal excursion (pHLE) ratings were completed prior to instrumental assessment. For these ratings, the SLPs used their index and middle fingers, with the center of the middle finger on the thyroid notch (Adam’s apple) and the index finger directly above (Fig. 3).

**Fig. 1:**
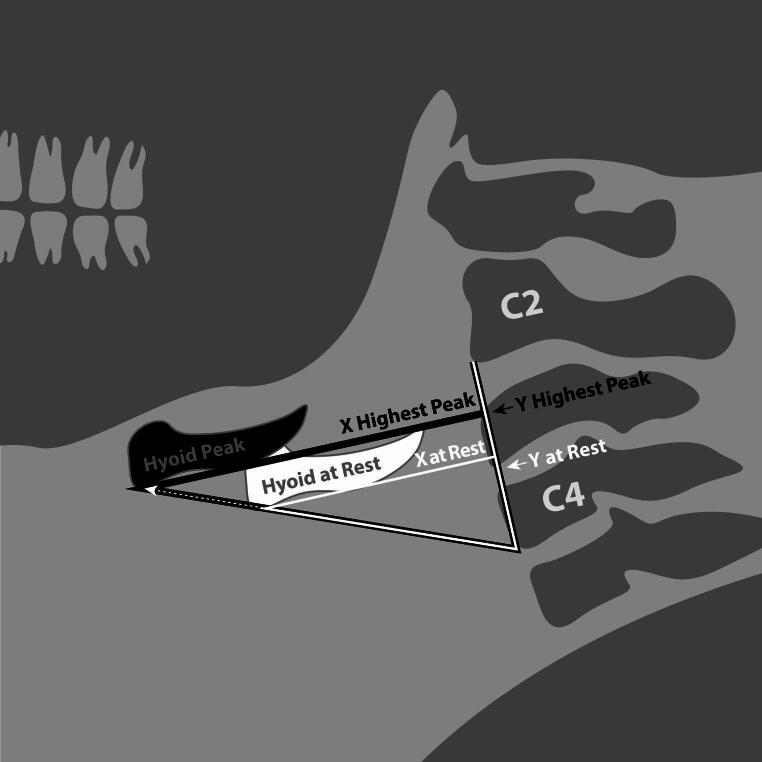
Example of spatial hyolaryngeal excursion at rest and at highest peak

**Fig. 2:**
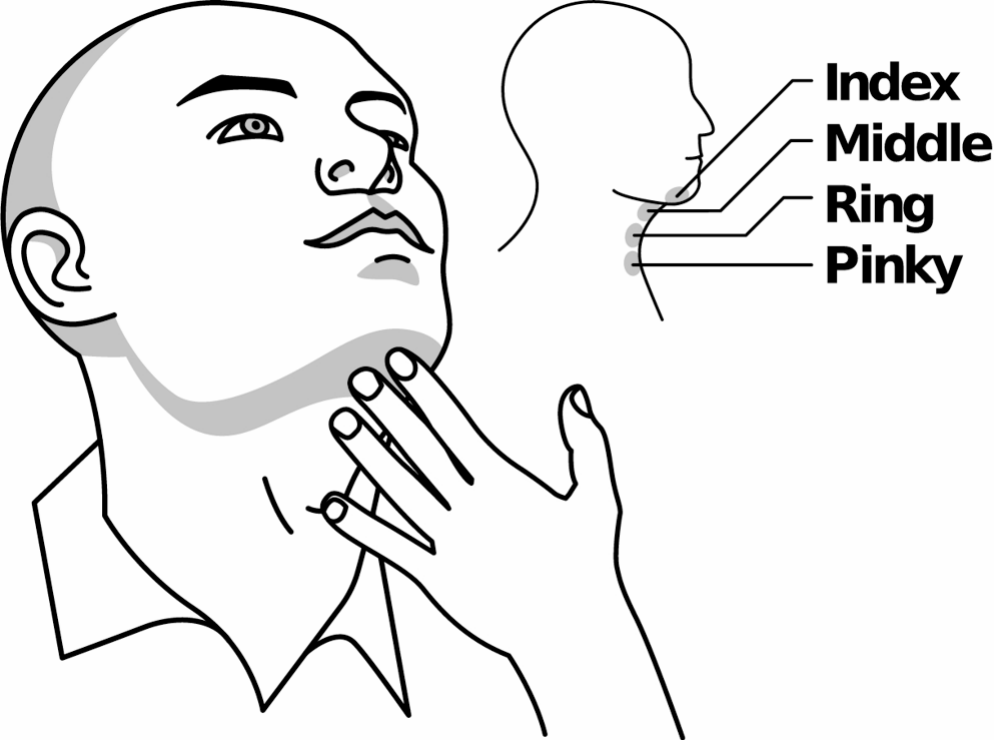
Example of assessing palpated HLE using Logemann’s four-finger palpation placement

**Fig. 3:**
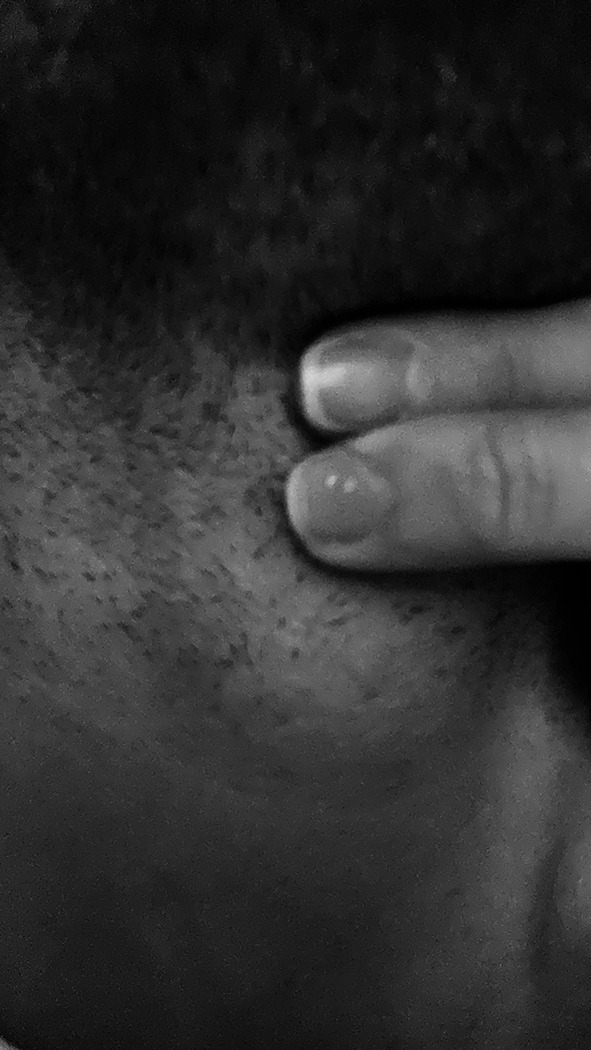
SLP’s two-finger placement for palpated HLE

Palpated HLE ratings were completed on up to three trials of 10 milliliters (ml) thin liquid (water) in 1 ounce medicine cups and three trials of 10 mL Snack Pack© vanilla pudding (Conagra Brands) presented on plastic spoons. Syringes were used to dispense precise amounts for both consistencies to either the medicine cups or the spoons. Trials were self-administered when possible or by the SLP as needed. The order of thin and pudding consistencies was randomized to reduce the risk of a biased order effect for the SLP ratings during the clinical swallow evaluation. Bailout criteria included signs of aspiration (e.g., voice quality change (VQ), coughs, or throat clears) across two consecutive trials within a consistency, resulting in the third trial not being administered. As this study was primarily conducted during clinical time, other trials and consistencies were completed for clinical purposes following the research trials as warranted on an individual basis.

**Fig. 4:**
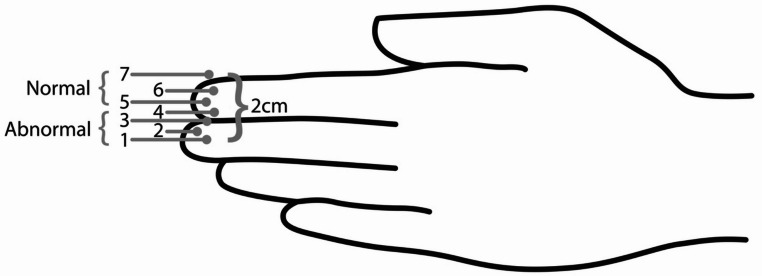
Measure and rating scale for palpated HLE

Elevation was defined as the maximum perceived distance of the palpated hyolaryngeal movement (via thyroid notch motion) during a swallow. Each trial received a separate pHLE rating by the SLP (defined below) (Fig. 4). In addition to pHLE ratings, other outcome measures that were documented following each trial were VQ, the presence of coughs or throat clears during or up to 15 s after the trial, and the number of swallows spontaneously completed for each trial. VQ was rated based on a solicited “ah” vocalization to document any potential changes from baseline vocal quality.

### Pilot Data and Development of pHLE Rating Training

Initial pilot data pHLE palpation ratings were completed using the SLP’s binary judgment of “normal” and “abnormal” pHLE. No specific definitions of normal and abnormal pHLE or training were provided to the raters during the pilot data collection, which reflects real-world clinical practice. Inter-rater reliability was completed on four patients across 24 pHLE ratings using the intraclass correlation (ICC) with the R package “irr” [30]. There was poor agreement among raters (ICC <.001, 95% CI [.-.554,.553], *p* =.05). Based on this poor agreement, pHLE definitions and rater training were developed and implemented to facilitate uniformity across raters to improve inter-rater reliability (Table 1).

**Table 1:** Palpated hyolaryngeal excurse (pHLE) definitions

Measure	Working pHLE definition
pHLE extent	SLP’s middle and index finger was measured from the center of the middle finger to the top of the index to determine where 2 cm fell per each SLP (Figs. 3 and 4). The rater placed the middle finger of their dominant hand directly on the thyroid notch with the index finger directly above the middle finger. Normal HLE movement of 2.1 cm during a healthy swallow (Kirita & Omura, 2015; Leonard et al., 2019) suggests that the thyroid notch should lift to the center of the index finger above or higher to be classified as normal. If the rater felt the thyroid notch lift from the center of the middle finger to the center of the index finger (or higher), the pHLE extent was rated as normal. Any elevation movement that was below the center of the index finger was rated as abnormal.

Previous work on spatial hyoid measures was used to develop formal palpation rating definitions and training. Normative data indicates an average of 2.1 centimeters (cm) of superior hyoid displacement during swallowing in non-dysphagic individuals [31], while hyoid elevation that is less than 1.5 cm is considered reduced [31, 32]. This typical displacement is comparable to the average width of an adult finger (2 cm +/- 0.24) [33]. For each SLP rater, it was verified that the distance from the center of the middle finger to the top of the adjacent index finger was approximately 2 cm (Fig. 4). A numerical rating scale was developed to capture the specific HLE displacement felt during palpation with each point on the scale representing approximately 25% of the width of a finger (Fig. 4). Position 5 on the scale was recognized as approximately 1.5 cm from position 1 and was used as the cut-point for “normal” pHLE. The raters were instructed to document the highest extent of pHLE felt during palpation, and that rating was used to categorize pHLE palpation extent as normal (categorized 5–7) or abnormal (categorized 4 or below) (Fig. 4).

Once the definitions and palpation rating scale were developed, training was completed across ten study patients using consensus rating to establish uniformity among the raters. Once the reliability with consensus reached good agreement (0.8), the SLPs continued to collect data as described without consensus. Interrater reliability checks were completed on ~ 20% of the sample (*n* = 17/87) with a second blinded SLP repeating two trials of 10mL thin and two trials of 10mL pudding with the patient and documenting pHLE ratings. Only patients who did not require a bailout during the CSE with the first SLP rater were included in reliability checks to limit the potential risk of aspiration.

### Videofluoroscopy Swallow Study (VFSS) Setup and Procedure

The setup for the VFSS included three trials of 10 mL of Varibar Thin Liquid barium sulfate (40% w/v) in 1 ounce medicine cups and three trials of 10 mL Varibar Pudding barium sulfate (40% w/v) on plastic spoons. Syringes were used to dispense precise amounts for all consistencies to either the medicine cups or the spoons. While three trials per consistency were prepped, the third trial was used only if deemed clinically appropriate by the SLP to reduce radiation exposure and allow time for other trials and consistencies that were completed for clinical purposes as warranted on an individual basis during the VFSS. To prioritize patients’ swallow safety during the VFSS, the trial presentation order was not randomized but determined using clinical decision-making, with the most common order including trials of 10 mL barium thin liquid prior to the 10 mL barium pudding trials. There were no pre-determined bailout criteria during VFSS; rather, the SLPs used their clinical expertise to determine when to stop specific consistency trials.

VFSSs were completed in the lateral view with patients seated upright in a Hausted VideoFluoroscopic Imaging Chair using a Siemens AXIOM Luminos TF fluoroscopy system with the pulse rate at 30 pulses per second [34]. Images were recorded at 30 frames per second using a Pentax 7245 C digital swallowing workstation (Pentax Medical, USA). While every attempt was made to capture multiple trials of each consistency, a patient was included for analysis purposes even if only one trial of a consistency was captured. It is worth noting that in clinical settings, there are instances where only one trial is completed for safety purposes, and SLPs are expected to use clinical decision-making skills given the limited trials.

### VFSS Spatial Analysis

The de-identified VFSS videos were clipped into individual trials so that each video file represented one bolus swallow. Each video clip was reviewed for hyoid peak positions. All video measures were completed in ImageJ (National Institutes of Health, https://imagej.nih.gov), using real-time and frame-by-frame analysis.

### Hyoid Peak Positions

Measures of HLE peak positions were taken from the VFSS, representing the maximum anterior and superior movements of the hyoid bone during swallowing. An anatomical scalar method was used to control individual height differences by reporting all distance-based measurements as relative to the size of the individual’s C2 to C4 vertebrae [35]. The frame with the greatest superior hyoid movement associated with swallowing was selected as the frame of interest for all the hyoid peak measurements. To ensure the frame with the highest superior movement was correctly identified, one frame before and one frame after the identified frame were also measured, and the numerical superior HLE peak across the three consecutive frames was selected. Peak anterior hyoid movement, referenced as X C2-C4%, follows HLE movement along the horizontal x-axis. Peak superior hyoid movement, referenced as Y C2-C4%, follows HLE movement along the vertical y-axis. An Excel spreadsheet was accessed from the Swallowing Rehabilitation Laboratory (Steele Swallowing Lab © 2022) for calculating X C2-C4% and Y C2-C4% (see appendix A).

## Spatial Measures Training and Consensus Rating

Consensus rating on VFSS data occurred on every trial following an approach comparable to that outlined in Steele [35] with trained research assistants selecting the VFSS frame of interest and completing the selected spatial measures. Raters were blinded to pHLE ratings for the same particiapnts, evaluation outcomes, and patient medical history. To meet consensus, there could be no more than one frame of difference between the frames of interest selected by rater pairs. Additionally, for all spatial measures completed by rater pairs, the ratio of the larger value to the smaller value could not exceed a value of 1.6. When consensus was not met, consensus meetings were completed to reach an agreement. When consensus was reached, but the ratings differed, the frame or measure with the largest superior peak position (Y) was selected. This is comparable to the approach used for a retrospective study on the same measures [17]. A flowsheet is provided outlining the setup and procedure for both the CSE and VFSS (see appendix B).

### Power Analysis

An ongoing power analysis with pilot data, G*Power 3.1, determined a target sample size of 78 would achieve a power of 0.08 and an alpha of 0.05 in the superior direction of HLE [36]. Therefore, the new target goal was to recruit 39 patients with normal pHLE and 39 with reduced pHLE to collect a diverse and representative sample. The initial power analysis used Brates [17] retrospective data to determine a target sample size of 104 would achieve a power of 0.08 and an alpha of 0.05 in the superior direction of HLE, with the initial target goal of 52 patients per group.

### Statistical Analysis

Descriptive data were summarized and analyzed statistically using R statistical software [37]. Interclass correlations (ICC) were repeated to determine inter-rater reliability among the SLP raters following training, using the R package “irr” [30]. Backwards regression procedures using the ‘stats’ package [37] were run to determine which of the independent variables best predicted either anterior (X) or superior (Y) position. The ‘lme4’ and ‘lmerTest’ packages [38, 39] were used to construct multiple linear regression models and determine the best-fitting model for each outcome variable (anterior and superior positions).

## Results

### Patients

Eighty-seven patients consented to the study. Due to imaging errors (e.g., points of interest were not viewable), data from 10 patients were excluded from the analysis. Seventy-seven patients were included in the final analyses (44 female, average age 71.6 years, age range: 28–96). Seventy-one patients were outpatient and 6 were inpatient (acute care). General primary etiologies included neurological/neuromuscular, structural, gastrointestinal, and/or respiratory disorders. See Table 2 for diagnoses per palpated hyolaryngeal excursion (pHLE) rating, noting some patients had more than one diagnosis. Fifty-one patients were rated as having normal pHLE palpation (26 female, average age 67.6, age range: 28–92) and 26 patients were rated as having reduced pHLE palpation (18 female, average age 77.4, age range: 58–96). Spatial hyolaryngeal excursion determined by instrumentation (iHLE) descriptives by anterior (X %C2-C4) and superior (Y %C2-C4) based on pHLE palpation rating are in Table 3, while iHLE by anterior (X %C2-C4) and superior (Y %C2-C4) based on bolus consistency and number of swallows (1 or ≥ 2) are in Tables 4 and 5, respectively.

**Table 2:** Diagnoses per palpation rating

	pHLE Normal *n* = 51	pHLE Abnormal *n* = 26		All participants *n* = 77
Neurological/Neuromuscular:	34/51 (67%)		19/26 (73%)	53/77 (69%)
Structural:	4/51 (8%)		7/26 (27%)	11/77 (14%)
Gastrointestinal (GI) Disorders:	19/51 (37%)		10/26 (38%)	29/77 (38%)
Respiratory:	15/51 (29%)		6/26 (23%)	21/77 (27%)

some participants had more than one diagnosis

**Table 3:** Descriptives statistics of Spatial measures of hyolaryngeal excursion (iHLE) per palpation rating

iHLE Measures (%C2-C4)	Palpation Rating	
	Rated Normal	Rated Abnormal
Peak Anterior (X %C2-C4) Mean (SD)	135.2 (18.8)	141.8 (24.5)
Peak Superior (Y %C2-C4) Mean (SD)	102.7 (25.2)	87.6 (35.8)

**Table 4:** Descriptives statistics of iHLE measures per bolus consistency

oHLE Measures (%C2-C4)	Bolus Consistency	
	Thin liquid	Puree
Peak Anterior (X %C2-C4) Mean (SD)	136.9 (21.9)	138.1 (20.3)
Peak Superior (Y %C2-C4) Mean (SD)	98.0 (30.4)	96.9 (29.3)

**Table 5:** Descriptives statistics of iHLE measures per number of swallows

oHLE Measures (%C2-C4)	Number of Swallows	
	1	≥ 2
Peak Anterior (X %C2-C4) Mean (SD)	136.7 (20.9)	138.7 (21.7)
Peak Superior (Y %C2-C4) Mean (SD)	98.2 (29.6)	96.3 (30.4)

### Reliability

Inter-rater reliability for pHLE was assessed using interclass correlations (ICC), following the development of pHLE definition and consensus training. Inter-rater reliability was completed on ~ 20% of the data set (*n* = 17). The ICC showed excellent agreement, ICC = 0.96, 95% CI [0.942, 0.978], *p <* 0.001. Inter-rater reliability was also conducted on the numerical pHLE palpation ratings from the pHLE scale (Fig. 4) for the same 17 patients and showed good agreement, ICC = 0.87, 95% CI [0.793, 0.919], *p* < 0.001.

### Linear Modeling

Two backward elimination regression analyses were conducted to identify the best-fitting model for predicting iHLE spatial differences in the anterior (forward) and superior (upward) direction. For each of the two outcome variables, nine predictors were included (five independent predictors and four interactions), so that the most complex model included the following as fixed effects: pHLE rating, bolus consistency, vocal quality changes, cough/throat clear response, the number of swallows per trial (1 or ≥ 2), and interactions between each of the latter four factors and pHLE rating. Patient and trial number were included as random effects in the model since patients received 1–2 trials of each consistency depending on their clinical outcomes (e.g., overt aspiration). Only the results from the best-fitting models are reported.

#### Anterior iHLE (X C2-C4%) Regression Model

The backward procedure eliminated all predictors except the random effect of the patient, suggesting that none of the fixed effects included in the saturated model account for significant variance in the anterior (forward) position of iHLE. Thus, assumptions for the anterior (forward) iHLE peak position were not conducted because no predictor variable aided in predicting the anterior iHLE outcome.

#### Assumptions Superior iHLE (Y C2-C4%)

Assumptions for superior (upward) iHLE (Y C2-C4%) were completed using the following R statistical packages ‘MASS’, ‘ggeffects’, and ‘car’ [40–42]. Assumptions checked for any violations for the best-fit models. No assumptions, including homoscedasticity, multicollinearity, independence of errors, and the test of normality, were violated.

#### Superior iHLE (Y C2-C4%) Regression Model

The backwards procedure determined that the best-fitting model for predicting superior (upward) position of iHLE included three independent predictors, in addition to the random, fixed effects of patient and trial number: pHLE palpation (Estimate = 11.2, SE. = 3.1, *p* = 0.0004), bolus consistency (Estimate = 2.5, SE. = 1.0, *p* = 0.01), and the number of swallows completed (1 or ≥ 2) during the CSE (Estimate = −4.4, SE. = 1.6, *p* = 0.007). Therefore, the independent factors that predict a significant increase in the superior position of iHLE included patient delineation of “normal” pHLE palpation (vs. “abnormal”), thin liquid trials (vs. puree trials), and trials involving a single swallow (vs. those involving more than one swallow) (Table 6).

**Table 6:** Summary of output from best-fitting statistical model for predicating superior kinematic measures iHLE from palpated HLE

Fixed factor	Estimate	SE	t value	*p* value
(Intercept)	90.3	3.9	23.9	< 0.001***
HLE palpation rating	11.2	3.1	3.6	0.0004***
Bolus Consistency	2.5	1.0	2.4	0.015*
Number of Swallows	−4.4	1.6	−2.7	0.007**

***significance < 0.001, **significance < 0.01, * < 0.05

## Discussion

This study aimed to determine if palpation could accurately predict reduced hyolaryngeal excursion (HLE). The results demonstrate that palpated HLE (pHLE) palpation can predict differences in spatial HLE measures determined by instrumentation (iHLE) for superior (upward) movement but is not sensitive to differences in anterior (forward) iHLE movement. Additionally, this study introduced that reliability for pHLE palpation is likely improved with standardized definitions and training for the SLP raters.

These findings prospectively confirm and are consistent with Brates, who also identified a link between retrospectively collected clinical HLE ratings and superior iHLE measures. Clinically, this demonstrates that speech-language pathologists (SLP) may be able to accurately identify reduced hyolaryngeal *elevation* during swallowing. As previously discussed, HLE is critical for airway protection by facilitating epiglottic inversion as part of laryngeal vestibule closure to prevent penetration and aspiration [5–8]. HLE also contributes to swallowing efficiency via pharyngoesophageal segment (PES) opening, allowing the bolus to pass into the esophagus, clearing the pharynx, and protecting the airway from post-swallow aspiration [5–7, 9, 10].

Palpated HLE that does not exceed a 1.5-centimeter (cm) span across fingers (approximately from the center of the middle finger to the center of the index finger, Fig. 4) may indicate reduced HLE. The findings also suggest that the number of swallows a patient uses on a single bolus trial may provide insight into the extent of superior HLE and inform the clinical decision-making process. SLPs reported in a recent survey that counting the number of swallows per bolus contributes to their interpretation of HLE during a clinical swallow evaluation (CSE) [20]. In the current study, when a patient swallowed more than once per bolus presentation, hyolaryngeal elevation was likely to be reduced. Multiple swallows may indicate pharyngeal residue, potentially related to reduced HLE [43–45]. Therefore, palpating multiple swallows on a single bolus may further confirm the potential for HLE abnormality and support the need for instrumental evaluation. This evidence reinforces the description and purpose of the CSE, highlighting its role in assessing swallowing function and identifying potential abnormalities [1]. The clinical evaluation is not just a pass/fail screening tool; it is a comprehensive diagnostic tool that requires critical thinking to integrate and interpret findings from various components and develop an appropriate plan of care.

The other predictor variable this study identified for HLE was bolus consistency. Patients were likely to have a higher spatial measure of HLE (i.e., better movement) with thin liquids than with puree consistencies. This finding reflects Steele [46] report of different iHLE values by consistency. Clinically, this may suggest that SLPs would benefit from different expectations from thin versus puree swallow HLE, both when palpating and during VFSS.

The superior regression model (y-axis) did not account for vocal quality changes or cough response to be predictors of reduced iHLE. Previous studies have identified that vocal quality changes (e.g., wet voice) are not a predictor of penetration or aspiration [47–50] and this study further confirms those findings. However, cough response is known to be highly related to dysphagia, along with other factors such as dysphonia, dysarthria, and failing the 3-oz swallow [16, 51]. Nevertheless, it warrants pointing out that cough response is not necessarily known to be directly associated with HLE.

Training the four SLP raters on an operational pHLE rating scale improved inter-rater reliability from poor (*n* = 4) to excellent (*n* = 17). Previous research has reported poor inter-rater reliability among SLP raters when they were not provided with training or working definitions to classify normal versus abnormal pHLE [52]. Other work predicting aspiration based on perceptual voice quality has demonstrated that rater training enhances reliability [47]. While relying on an informal individualized rating system replicates likely real-world HLE palpation practice, these judgments were too varied and may have interfered with the ability to answer the research question. Based on pilot data and referenced pHLE norms, the current study developed an internal rating scale for HLE palpation and completed training with the SLP raters (Table 1). These definitions likely helped improve accuracy among the SLPs as they were using the same outlined scale instead of relying on an individualized approach that likely differed from rater to rater. The training process facilitated an excellent agreement between the SLP raters in judging normal versus abnormal pHLE extent measures.

While these results may add to the interpretation of CSE components, it remains essential for healthcare professionals to continue to access instrumental swallowing assessment for a comprehensive understanding of the oropharyngeal physiology before initiating treatment [1, 53]. Treatment decisions, including diet modification, appropriate compensatory strategies, and/or rehabilitation recommendations, should be based on the underlying physiological impairments instrumentally observed [54]. The findings from this study provide SLPs with methods to improve the utility of certain pHLE judgments, which may, in turn, improve CSE interpretations and appropriate instrumental referrals. We have shown that HLE palpation is interpretable for superior (upward) movement of HLE and the number of swallows per bolus, which can help rule in potential laryngeal elevation contributions to pharyngeal dysphagia.

In summary, the findings indicate that SLPs trained on using a 2 cm rule for judging HLE using palpation are likely reliable at discerning a difference in superior (upward) movement of HLE. To our knowledge, this is the first prospective and controlled study that demonstrates the clinical utility of HLE palpation. Further research is required to ascertain whether the reliability observed in this study can be maintained when standardization and training on palpation are applied across a more extensive sample of SLPs. Investigations focused on primary etiologies in isolation (i.e., more homogeneous patient samples) may reveal whether palpation is more beneficial for specific patient groups in guiding clinical decision-making and the subsequent plan of care. Future investigations might also explore the potential utility of palpation in perceptually evaluating other facets of iHLE movement, such as speed and timing. Lastly, while this study suggests that pHLE can predict superior iHLE measures, it does not ascertain whether palpation can also predict more functional pharyngeal stage consequences, such as aspiration or residue. Therefore, further research is warranted to refine the clinical utility of palpation in guiding clinical decision-making.

### Limitations

This study is not without limitations, including that all patients were referred for a swallow evaluation by a physician, meaning that all patients demonstrated signs and/or symptoms of dysphagia, which may have introduced selection bias. Thus, there is no control group of healthy patients without signs/symptoms of dysphagia. The number of patients judged as having normal pHLE was approximately double that of the reduced pHLE group, likely related to recruitment primarily occurring from outpatient patients. This may have limited the severity of the study sample’s dysphagia, which may be a reason cough was not a predictor of iHLE. Literature has shown that cough response can indicate incomplete airway protection [55, 56]. It is also possible that cough may be a predictor of iHLE in a specific and more acute etiology (e.g., stroke). The four SLP raters for this study all had approximately the same finger width between the middle and index fingers used for palpation, which made training more straightforward; however, it is anticipated that this is not representative of all SLPs, which could impact training and working definitions that were used for this study. This study did not investigate if the 2 cm palpation method impacted the SLPs’ cognitive load during the CSE assessment, which could impact the observation and interpretation of other dysphagia signs. Furthermore, this study assessed HLE via palpation in the coronal plane, a method that does not readily capture anterior excursion. Future research should explore sagittal-plane palpation as a potentially more effective approach for assessing anterior movement.

## Conclusion

In conclusion, assessing hyolaryngeal excursion through palpation during a clinical swallow evaluation is an effective method to identify potential decreases in the superior (upward) movement of the hyoid peak position. This provides evidence to support further investigation into the application of palpation for assessing HLE and its potential to improve diagnostic accuracy. Lastly, reliability amongst SLP raters improved when raters were trained to standard definitions of HLE adequacy.

## Supplementary Information

Below is the link to the electronic supplementary material.

Supplementary Material 1

Supplementary Material 2

## Data Availability

Data generated or analyzed during this study are not publicly available due to confidentiality agreements with research collaborators.
