# Supplementary material for: Palpation as a Method To Predict Spatial Instrumental Hyolaryngeal Excursion Measures
Source: Dysphagia. 2025 Sep 25;41(1):285–95. doi: 10.1007/s00455-025-10880-w (PMC12950094; doi:10.1007/s00455-025-10880-w)
Supplement: Supplementary file 1 — Supplementary Material 1 [file 455_2025_10880_MOESM1_ESM.docx]

**Appendix A:**

$$Kinematic Anterior Peak Position X:ABS(\left( \left( C4x-Hyoidx \right)\times COS\left( ATAN\left( \left( C4x-C2x)\div(C4y-C2y \right) \right) \right)-\left( C4y-Hyoidy \right)\times SIN\left( ATAN\left( \left( C4x-C2x \right)\div\left( C4y-C2y \right) \right) \right) \right)\div SQRT\left( \left( C4x-C2x \right)^{2}+\left( C4y-C2y \right)^{2} \right))\times100))$$

$$Kinematic Superior Peak Position Y: -(\left( C4x-Hyoidx \right)\times SIN\left( ATAN\left( \left( C4x-C2x \right)\div\left( C4y-C2y \right) \right) \right)+\left( C4y+Hyoidy \right)\times COS\left( ATAN\left( \left( C4x-C2x \right)\div\left( C4y-C2y \right) \right) \right))\div SQRT\left( \left( C4x-C2x \right)^{2}+\left( C4y-C2y \right)^{2} \right))\times100))$$
