# Supplementary material for: Palpation as a Method To Predict Spatial Instrumental Hyolaryngeal Excursion Measures
Source: Dysphagia. 2025 Sep 25;41(1):285–95. doi: 10.1007/s00455-025-10880-w (PMC12950094; doi:10.1007/s00455-025-10880-w)
Supplement: Supplementary file 2 — Supplementary Material 2 [file 455_2025_10880_MOESM2_ESM.docx]

**Appendix B:**

*CSE and VFSS process flowchart from setup through consensus*

*Note.**VFSS had up to three trials of each consistency
